# Lithium and Not Acetoacetate Influences the Growth of Cells Treated with Lithium Acetoacetate

**DOI:** 10.3390/ijms20123104

**Published:** 2019-06-25

**Authors:** Silvia Vidali, Sepideh Aminzadeh-Gohari, Renaud Vatrinet, Luisa Iommarini, Anna Maria Porcelli, Barbara Kofler, René Günther Feichtinger

**Affiliations:** 1 Research Program for Receptor Biochemistry and Tumor Metabolism, Department of Pediatrics, Paracelsus Medical University, 5020 Salzburg, Austria; s.vidali@salk.at (S.V.); s.aminzadeh-gohari@salk.at (S.A.-G.); r.feichtinger@salk.at (R.G.F.); 2 Department of Pharmacy and Biotechnology (FABIT), University of Bologna, 40126 Bologna, Italy; renaud.vatrinet@gmail.com (R.V.); luisa.iommarini2@unibo.it (L.I.); annamaria.porcelli@unibo.it (A.M.P.)

**Keywords:** lithium, acetoacetate, ketone bodies, cancer, ketogenic diet

## Abstract

The ketogenic diet (KD), a high-fat/low-carbohydrate/adequate-protein diet, has been proposed as a treatment for a variety of diseases, including cancer. KD leads to generation of ketone bodies (KBs), predominantly acetoacetate (AcAc) and 3-hydroxy-butyrate, as a result of fatty acid oxidation. Several studies investigated the antiproliferative effects of lithium acetoacetate (LiAcAc) and sodium 3-hydroxybutyrate on cancer cells in vitro. However, a critical point missed in some studies using LiAcAc is that Li ions have pleiotropic effects on cell growth and cell signaling. Thus, we tested whether Li ions per se contribute to the antiproliferative effects of LiAcAc in vitro. Cell proliferation was analyzed on neuroblastoma, renal cell carcinoma, and human embryonic kidney cell lines. Cells were treated for 5 days with 2.5, 5, and 10 mM LiAcAc and with equimolar concentrations of lithium chloride (LiCl) or sodium chloride (NaCl). LiAcAc affected the growth of all cell lines, either negatively or positively. However, the effects of LiAcAc were always similar to those of LiCl. In contrast, NaCl showed no effects, indicating that the Li ion impacts cell proliferation. As Li ions have significant effects on cell growth, it is important for future studies to include sources of Li ions as a control.

## 1. Introduction

A growing number of preclinical and clinical studies reports anticancer effects of dietary intervention by the ketogenic diet (KD), a high-fat, low-carbohydrate, and moderate-protein diet. Preclinical evidence strongly suggests that manipulation of metabolism by the KD may be an effective therapy against certain types of tumors and may actually enhance the efficacy of current standard therapies for cancer [1]. However, the mechanisms underlying the antitumor benefits of the KD are not yet fully understood. Part of the therapeutic effect induced by the KD is assumed to be achieved by the production of ketone bodies (KBs) and subsequent mimicking of a fasting condition [2]. KBs are water-soluble molecules produced in the liver as a result of fatty acid oxidation during times of fasting, starvation, prolonged intense exercise, or carbohydrate restriction (such as caused by the KD). The most abundant and relevant KBs are acetoacetate (AcAc) and 3-hydroxy-butyrate (3HB). Acetone, a third KB, is a spontaneous breakdown product of AcAc [3,4].

In vivo experiments showed that ketosis induced by the KD negatively correlates with tumor growth and increases the efficacy of chemotherapy [1,5]. Other studies indicate that the KD simultaneously targets multiple features of cancer, including its altered energy metabolism, associated inflammation, hypoxia, angiogenesis, and tissue invasion [2,4]. However, it is not clear whether KBs directly contribute to the KD-related changes observed in tumors. To investigate how KD might selectively impair tumor cell growth, various in vitro studies have focused on the effects KBs may have on tumor growth (Table 1).

In the present study, we highlight an important issue that must be considered when designing in vitro and in vivo KB studies. At present, the most-used KBs in different experiments are lithium acetoacetate (LiAcAc) and sodium 3HB (NaHB). Studies have reported that the LiAcAc salt inhibits proliferation of different types of cancer cell lines, including human colon, breast, pancreatic, and neuroblastoma. Furthermore, LiAcAc has been used to study the effects of AcAc on signaling pathways and other cellular phenomena involved in cancer and proliferation (Table 1). A critical point in such studies is that Li itself might exert antineoplastic effects on cell growth and cellular signaling. However, of the studies listed in Table 1, less than half reported having used equimolar concentrations of lithium chloride (LiCl) as a control. Other studies focused instead on the function of 3HB only, which is usually used as a sodium salt, and therefore was not further investigated in the present study. Studies where sodium AcAc, ethyl AcAc or unspecified forms of AcAc was used have been excluded in this report.

The present work aimed to clarify whether the effect of LiAcAc on cell growth or tumor metabolism is due to the AcAc ion or is actually a result of the Li ion.

## 2. Results

### 2.1. Lithium Alone is Responsible for the Effects of LiAcAc on Cell Growth

Based on literature observations, we investigated if Li ions are responsible for the growth-inhibitory effects of LiAcAc. Cells were treated for 5 days with either LiAcAc or LiCl. Non-tumor human embryonic kidney (HEK)293 cells and normal human dermal fibroblasts (HDFn) were used as a control to investigate if the effects were specific to tumor cells.

**Figure 1:**
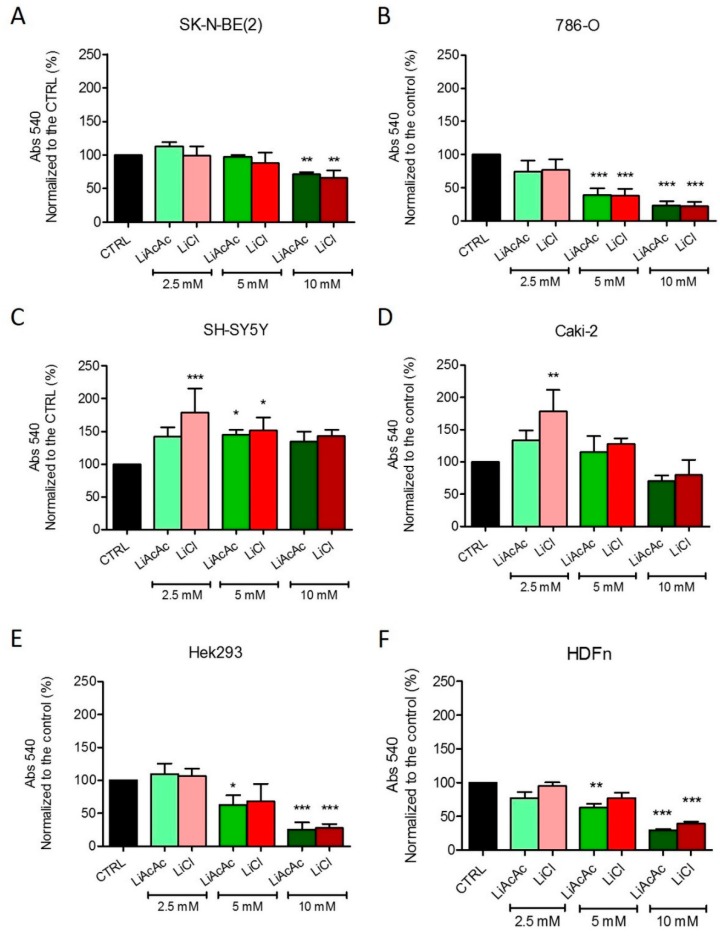
Growth of SK-N-BE(2) (**A**), 786-O (**B**), SH-SY5Y (**C**), CAKI-2 (**D**), HEK293 (**E**), and HDFn (**F**) cells treated with 2.5 mM, 5 mM, and 10 mM LiAcAc or LiCl. Data are given as mean ± SD. Statistical analysis was performed by using one-way ANOVA (Dunnett’s Multiple Comparison Test); treatments vs. control: * *p* < 0.05, ** *p* < 0.01, *** *p* < 0.001; *n* = 3 independent experiments (6 wells/treatment/experiment).

In the more aggressive neuroblastoma (NB) cell line SK-N-BE(2) and the more aggressive renal cell carcinoma (RCC) cell line 786-O, LiAcAC reduced cell growth in a dose-dependent manner, with the 10-mM concentration being the most inhibitory. Interestingly, LiCl exerted growth-inhibitory effects remarkably similar to those of LiAcAc (Figure 1A,B). Treatment of NB cell line SH-SY5Y with LiAcAc or LiCl promoted an increase in cell proliferation (Figure 1C). Treatment of RCC CAKI-2 cells elicited a response similar to that of SH-SY5Y cells (Figure 1D). Moreover, in the latter two cell lines, at the 2.5-mM concentration, LiCl showed a stronger effect compared to LiAcAc. Nevertheless, the effects of the 5-mM and 10-mM concentrations of LiCl matched the effects of LiAcAc at the corresponding concentrations (Figure 1C,D). Interestingly, the HEK293 cells and the HDFn responded to LiAcAc or LiCl exactly like the more aggressive tumor cell lines, namely an absence of an effect at the 2.5-mM concentration and growth inhibition at the two higher concentrations (Figure 1E,F). Interestingly, similar results were obtained using the sulforhodamine B (SRB) assay, indicating that the effects on proliferation are independent from the assay used.

### 2.2. Chloride Does Not Affect Cell Growth

**Figure 2:**
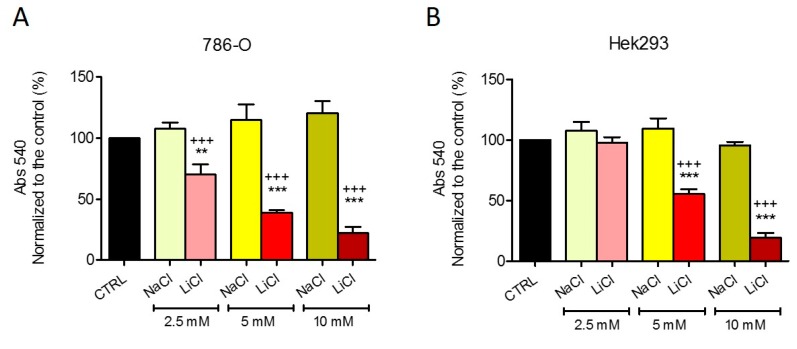
Growth of 786-O (**A**) and HEK293 (**B**) cells treated with 2.5 mM, 5 mM, and 10 mM LiCl or NaCl. Data are given as mean ± SD. Statistical analysis was performed by using one-way ANOVA (Dunnett’s Multiple Comparison Test); treatments vs. control: ** *p* < 0.01, *** *p* < 0.001; LiCl vs. equimolar NaCl: ^+++^ *p* < 0.001; *n* = 3 independent experiments (6 wells/treatment/experiment).

To exclude any influence of the chloride ion from the LiCl salt, we performed a control experiment with NaCl. 786-O cells and HEK293 cells were treated with equimolar concentrations of LiCl or NaCl for 5 days. The antiproliferative effects of LiCl were, again, similar to those observed in Figure 1, whereas NaCl did not exert any significant effect (Figure 2), confirming that the Li ion is responsible for the observed effects on cell growth. In all experiments, when media was refreshed, there were no dead cells floating, suggesting that the decreased cell viability is attributable to an antiproliferative effect rather than to increased cell death.

### 2.3. LiCl and LiAcAc Do Not Increase the Expression of Cleaved Caspase 3

**Figure 3:**
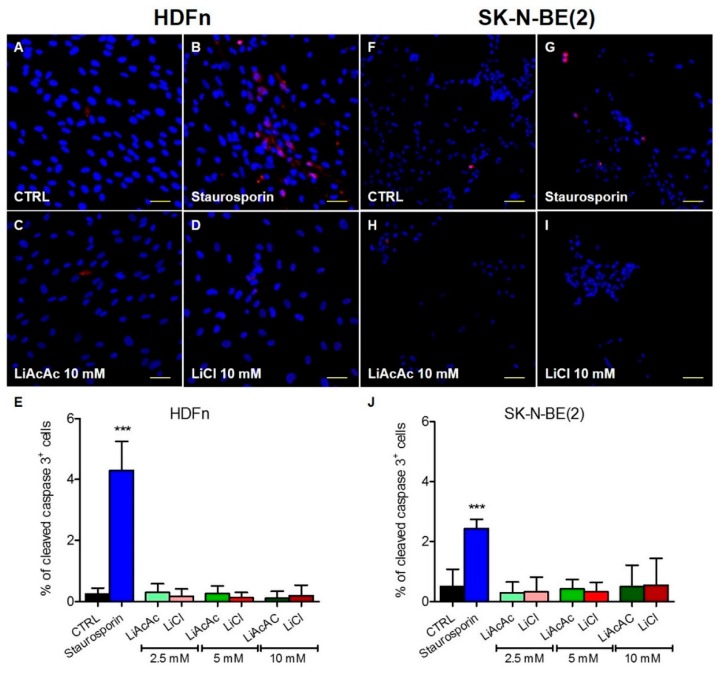
Immunofluorescence staining of cleaved caspase 3 (red): HDFn (**A**–**D**) and SK-N-BE(2) (**F**–**I**) cells were treated with 2.5, 5, and 10 mM LiAcAc (**C**,**H**) or equimolar concentrations of LiCl (**D**,**I**) for 5 days. As positive control, cells were treated for 3 hours with staurosporin 1 µM (**B**,**G**). Nuclei are demarcated with DAPI (blue). Scale bar: 50 µM. Figure 3 (**E**,**J**) shows the percentage of cleaved caspase 3 positive HDFn (**E**) and SK-N-BE(2) (**J**) cells. Data are given as mean ± SD. A statistical analysis was performed by using one-way ANOVA (Dunnett’s Multiple Comparison Test); data are compared to untreated control cells: *** *p* < 0.001; *n* = 9–10 macroscopic fields.

To investigate if LiAcAc or LiCl influence cell death, cleaved caspase-3 staining was performed on two selected cell lines. HDFn and SK-N-BE(2) were selected because they both showed a consistent cell growth inhibition when treated with 10 mM LiAcAc or LiCl. None of the tested concentrations of LiAcAc and LiCl increased the percentage of cleaved caspase 3 positive cells. On the contrary, the cells treated with 1-µM staurosporin for 3 h as positive control for induction of apoptosis revealed a significant increase of apoptotic cells shown by increased levels of cleaved caspase 3 positive cells (Figure 3). These results suggest that LiCl and LiAcAc do not increase cell death by apoptosis at the tested doses and time points.

## 3. Discussion

Over the past decade, there has been increased interest in the metabolic alterations that attend cancer and how they might be targeted therapeutically. Aerobic glycolysis and reduced oxidative phosphorylation are two such well-identified metabolic alterations in cancer cells [16], a phenomenon called the Warburg effect [17].

The KD and direct supplementation with KBs have been considered as part of a multimodal therapeutic arsenal against cancer to target the Warburg effect [4]. The initial intent of applying the KD in cancer therapy was to deprive the body of glucose and to force it into ketosis so that it uses KBs as alternative sources of energy [4].

In vitro experiments with KBs are a first step in the evaluation of the mechanism of KD therapy in cancer; however, the fact that commercially available KBs are usually salt compounds is potentially problematic for in vitro analyses of KB effects.

Acetoacetate (AcAc), one of the two principal KBs in blood, is often used in research in the form of LiAcAc salt in both in vitro and in vivo experiments (Table 1). Studies have also provided some plausible molecular mechanisms by which AcAc could affect the growth of cancer cells versus normal cells, such as the inhibition of ATP production and the promotion of apoptosis of cancer cells (Table 1). In the same context, our results show that LiAcAc inhibits cell growth in a dose-dependent manner in three of the five cell lines we tested, especially at the highest (10 mM) concentration. However, we also observed that LiCl induced very similar results as LiAcAc. In agreement with our findings, several publications report that Li inhibits cancer cell growth [18,19,20] and/or enhances the efficacy of standard cancer therapies [21,22] both in vitro and in vivo.

The reason for the different response between the different cell lines is unknown. However, we hypothesize that cancer cells with higher cell division rates could be more susceptible to the growth inhibition provoked by lithium. This ion can regulate cell function and metabolism at different levels, from activation of antiapoptotic proteins to inhibition of several transcription factors and modulation of redox balance and mitochondrial function [23]. Moreover, at therapeutic doses, lithium displays neuro-, cardio-, and nephroprotective properties [24,25]. Usually, cells derived from less aggressive tumors are less differentiated from the cells from which the tumor originated. More aggressive tumors tend to lose most of the original identity. Moreover. the cells tested in this work are of neuro- or nephro-origin, which could explain why, at lower doses, LiCl showed stimulatory effects in the less aggressive tumor cell lines CAKI-2 and SH-SY5Y and no effects or disruptive effects in the more aggressive ones, 786-O and SK-N-BE(2). The fact that, at higher doses, non-tumor cells are also affected in terms of cell growth highlights the necessity that any drug containing Li has to be properly dosed in clinical therapy.

Due to the multipotent effects of Li on different cellular processes, misinterpretation of data from experiments with LiAcAc might extend beyond cell-growth studies [26]. For instance, Li markedly affects different aspects of mitochondrial function, including mitochondrial biogenesis, ATP generation, and production of reactive oxygen species [27,28,29].

Moreover, several studies have reported that Li activates nuclear factor erythroid 2-related factor 2 (NRF2), a key transcription factor that regulates the expression of antioxidant proteins in response to oxidative stress [30,31]. Similarly, activation of the NRF2 pathway has also been reported in KB-treated endothelial cells, although the authors did not clarify whether that activation resulted from LiAcAc, NaHB, or both KBs [12].

Interestingly, in multiple myeloma cells, LiCl is able to reduce the amount of cells in G1 phase in a dose-dependent manner and to induce G2 cell cycle arrest and apoptosis at doses of 10 mM or higher [32]. These results are in line with our hypothesis that, at least at lower doses, lithium does not cause cell death but influences cell cycle arrest.

Not all effects of LiAcAc are due to Li, however. For example, a recent study analyzed the effects of LiAcAc administered intraperitoneally to mice. In the study, LiCl was used as a control and had no effects on the parameters tested, proving that the effects of LiAcAc were all attributable to AcAc [7]. Although this work is focused on cell growth and metabolism, it is important to point out that LiAcAc has been used also in several other studies where the authors focused on other parameters. Some of the studies also used LiCl a control [33,34], but several others studies did not [35,36]. Most importantly, there are reports where the authors do not specify which type of AcAc salt has been used, making it difficult to evaluate if the results are based on proper controls [37,38,39].

Taken together, only a few studies have given more than nominal attention to the potential cell growth-inhibitory effect of Li when using LiAcAc (Table 1). Based on our findings and those of other studies, we argue that using LiAcAc without a corresponding control could lead to misinterpretation of the data and erroneous conclusions. To avoid this, we suggest, when using AcAc, also use LiCl as a control. Alternatively, other AcAc derivatives such as ethyl AcAc (with concerns of the effects of the ethyl group per se) or sodium AcAc could be used [40,41]. Our study also highlights the need for chemical suppliers to offer the Na-conjugated form of AcAc, which currently is not available from most major chemical companies.

## 4. Material and Methods

### 4.1. Cell Culture

All media were supplemented with heat-inactivated fetal bovine serum (Gibco, Vienna, Austria) and penicillin/streptomycin amphotericin B solution (Lonza, Cologne, Germany).

All cell lines have been purchased from American Type Culture Collection. NB cell lines SH-SY5Y and SK-N-BE(2) were cultured in a 1:1 mixture of Ham’s F-12 medium (Sigma-Aldrich, Austria) and Eagle’s minimum essential medium (Sigma-Aldrich, Vienna, Austria). The mixture was supplemented with GlutaMAX (Gibco, Vienna, Austria) and MEM nonessential amino acid solution (Sigma-Aldrich).

RCC 786-O cells were cultured in a high glucose RPMI-1640 medium (Sigma-Aldrich), and RCC CAKI-2 cells were grown in McCoy’s 5a medium (Sigma-Aldrich).

The human embryonic kidney cell line HEK293 and the human normal dermal fibroblasts (HDFn) were grown in high glucose Dulbecco’s modified Eagle’s medium DMEM (Sigma-Aldrich), supplemented with GlutaMAX.

### 4.2. Crystal Violet Assay

Cell viability was measured by crystal violet staining. Cells were seeded in 96-well plates (1 × 10^3^ cells/well for the two RCC, the HEK293, and the HDFn cell lines and 1.5 × 10^3^ cells/well for the NB cell lines). After 24 h, the cultures were supplemented with 2.5 mM, 5 mM, and 10 mM LiAcAc (Sigma-Aldrich), LiCl (Alfa Aesar, Kandel, Germany), or NaCl (Merck, Vienna, Austria). The control groups (CTRL) were treated with cell culture media alone. All substances were freshly dissolved in culture media prior to the start of each experiment. Every second day, the media were changed and fresh salts were added. The total length of the treatments was 5 days.

At the end of the treatment, the medium was removed from each well, 50 µl of 3.7% paraformaldehyde were added, and the samples were incubated at room temperature for 15 min. After removing the paraformaldehyde, 200 µL of a 0.05% crystal violet (Sigma-Aldrich) filtered solution was added and the samples were incubated at room temperature for 30 min. After removal of the crystal violet solution, the wells were washed 3 times with tap water. The plates were inverted and air-dried for 30 min. In each well, 300 µL of methanol were added to dissolve the dye and, after gentle shaking, the plates were read by spectrophotometry at 540 nm.

### 4.3. Immunohistochemical Staining of Cleaved-Caspase 3

Immunohistochemical staining was performed as previously described [42]. Briefly, HDFn and SK-N-BE(2) cells were grown on coverslips and treated with LiCl and LiAcAc as for the proliferation assay.

Treatment with staurosporin 1 µM for 3 h was used as a positive control of apoptosis. After 24 h fixation in 4% formalin, samples were incubated in antigen retrieval buffer for 40 min at 95 °C. Slides were incubated for 1 h at RT with the primary antibody (polyclonal rabbit anti-cleaved caspase 3 (1:200, Cell Signaling, Frankfurt am Main, Germany) diluted in Dako antibody diluent with background reducing components (Dako, Vienna, Austria). Slides were incubated with the secondary antibody Alexa-Fluor-594-conjugated donkey anti-rabbit IgG (1:1000, 1 h, room temperature, Invitrogen, Vienna, Austria) diluted in PBS-T. Coverslips were incubated 10 min in a DAPI solution in PBS-T. Finally, coverslips were washed in ddH_2_O and mounted with s fluorescent mounting medium (Dako, Vienna, Austria). Microscopy was carried out with a Zeiss LSM 880 confocal microscope.

### 4.4. Statistical Analysis

Statistical analysis was performed using Prism 6 (GraphPad Software, San Diego, CA, USA). All results are given as mean ± SD. One-way ANOVA (Dunnett’s Multiple Comparison Test) was used for determination of significance.

## Figures and Tables

**Table 1:** In vitro and in vivo studies investigating the effects of LiAcAc on cell proliferation, tumor growth, and cancer metabolism.

Effect of LiAcAc	Equimolar Controls	Ref.
LiAcAc or LiCl (1.5 mM) did not affect proliferation in breast cancer cells.	LiCl	[6]
LiAcAc but not LiCl increased BRAF positive melanoma tumor growth and increased MEK1 and ERK1/2 phosphorylation in the same cells. No effects is shown in BRAF negative melanomas.	LiCl	[7]
Both LiAcAc and LiCl (10 mM) inhibited bovine lymphocytes proliferation; the 3.125 mM concentration showed a trend to enhance the proliferation.	LiCl, NaCl	[8]
LiAcAc but not LiCl (3.6 mM) enhanced development of bovine embryos. Higher concentrations of both compounds were inhibitory.	LiCl	[9]
LiAcAc and LiCl (5 mM) neither affected glioma cells growth in normoxia nor in hypoxia conditions.	LiCl	[10]
LiAcAc (10 mM) significantly reduced cell growth and ATP and UCP2 production in several colon and breast cancer cell lines.	n.r.	[11]
LiAcAc (0.5–5 mM) reduced viability in dermal endothelium cells. A solution of 4 mM 3-HB and 1 mM LiAcAc induced moderate oxidative stress and reduced Nrf2 expression.	n.r.	[12]
LiAcAc (10–50 mM) reduced mouse hippocampal neuronal cell growth. LiAcAc (1–5 mM) protected the cells from glutamate induced toxicity.	n.r.	[13]
LiAcAc (5–20 mM) reduced pancreatic cancer cell growth. LiAcAc (10 and/or 20 mM) increased caspase 3/7 activity and reduced glucose and glutamine uptake, lactate release, ATP and ROS levels, and the expression of glycolytic enzymes. LiAcAc (20 mM) prevented cachexia.	n.r.	[14]
LiAcAc (13.9 mM) did not prevent the decrease in cell viability in neuroblastoma cells deprived of glucose but increased apoptosis. Normal fibroblasts were not affected.	n.r.	[15]

ATP, adenosine 3-phosphate; BRAF, proto-oncogene B-Raf; ERK, extracellular-signal regulated kinases; MEK, Mitogen-activated protein kinase kinase; Nrf2, Nuclear factor (erythroid-derived 2)-like 2; n.r., not reported; Ref., reference; UCP, mitochondrial uncoupling protein.

## Abbreviations

3-HB	3-hydroxy-butyrate
AcAc	Acetoacetate
B3GALANT2	Beta-1,3-N-acetylgalactosaminyltransferase 2
HDFn	Normal human dermal fibroblasts
HEK	Human embryonic kidney
KB	ketone bodies
KD	ketogenic diet
NB	Neuroblastoma
RCC	Renal cell carcinoma
